# Evaluation of Refractive Status Changes Following Pterygium Surgery With the Bare Sclera Technique Versus Conjunctival Autografting in a Tertiary Eye Care Centre in Kolkata: A Prospective Comparative Study

**DOI:** 10.7759/cureus.96797

**Published:** 2025-11-13

**Authors:** Indrani Bhattacharyay, Somnath Das, Sahadev Santra, Sayan Bhoumik

**Affiliations:** 1 Ophthalmology, Regional Institute of Ophthalmology, Medical College and Hospital, Kolkata, IND; 2 Community Medicine, Vardhman Mahavir Medical College and Safdarjung Hospital, New Delhi, IND; 3 Radiodiagnosis, Prafulla Chandra Sen Government Medical College And Hospital, Arambagh, IND

**Keywords:** astigmatism, bare sclera, conjunctival autografting, keratometry, pterygium, visual acuity

## Abstract

Background

Therapeutic options for pterygium are primarily surgical. Several techniques are available for the surgical excision of pterygium. This study aims to compare the refractive status change following pterygium surgery between the bare sclera and the limbal conjunctival autograft technique.

Methods

The study subjects included 110 patients (110 eyes) with primary nasal pterygium who underwent surgery. After a full preoperative assessment, all patients were randomly allocated to either group A (bare sclera technique, BST) or group B (limbal conjunctival autograft technique, LCAT). All patients were assessed for visual acuity in LogMAR score, refraction in diopters (D), and keratometry values in diopters (D) at the first OPD visit and postoperatively after two weeks, four weeks, and 12 weeks. Quantitative data were expressed as means and standard deviations, while qualitative data were expressed in frequencies and percentages. To compare the variables, paired and unpaired t-tests were used. A p< 0.05 was considered statistically significant.

Result

The preoperative mean visual acuity was improved from LogMAR score 0.37±0.15 to LogMAR score 0.15±0.10 with a p-value <0.001 and from LogMAR score 0.37±0.17 to LogMAR score 0.08±0.09 with a p-value <0.001 after 12 weeks of surgery in the BST and the LCAT, respectively. The preoperative mean astigmatism was reduced from 2.10±0.84 D to 1.30±0.56 D (p <0.001) and from 2.08±0.96 D to 0.70±0.40 D (p <0.001) after 12 weeks of surgery in the BST and LCAT, respectively. The improvement in mean visual acuity was better in the LCAT than in the BST, and the difference between the groups was statistically significant (p <0.001) after 12 weeks of surgery. The reduction in post-operative mean astigmatism was greater in the LCAT than in the BST, and the difference between the groups was statistically significant (p < 0.001) after 12 weeks of surgery. The difference in keratometry values after 12 weeks of surgery between the two groups was also found to be statistically significant (p < 0.05).

Conclusion

Pterygium excision resulted in better improvement in the limbal conjunctival autograft technique than the bare sclera technique in terms of visual acuity, astigmatism, and keratometry values.

## Introduction

Pterygium (Latin for little wing) is an abnormal fibrovascular growth that starts from the bulbar conjunctiva, usually on the nasal side, and gradually extends onto the corneal surface [1]. The prevalence of pterygium varies globally, with evidence suggesting ultraviolet light as a major contributing factor [2]. Pterygium is commonly reported in India because of its tropical location [3]. Therapeutic options for pterygium are primarily surgical. The main indication for pterygium surgery is visual disturbance due to encroachment over the pupillary area or induced astigmatism, while other considerations include restriction of eye movements, chronic redness with foreign body sensation, and cosmetic concerns [4]. Astigmatism occurs due to the pooling of the tear film, the mechanical traction exerted on the cornea, and the size of the pterygium, and can be measured with the refraction technique, keratometry, and corneal topography [5]. This corneal astigmatism can be reduced with pterygium excision surgery, which involves the use of several techniques [6].

This study was conducted since only a few studies assessed the change in refractive status following pterygium surgery with the limbal conjunctival autograft technique (LCAT) compared to the bare sclera technique (BST). The objective of the study was to quantify the amount of refractive changes following pterygium surgery in primary nasal pterygium patients and then to compare the changes between the BST and the LCAT.

## Materials and methods

A prospective comparative study was conducted on a total of 110 patients (110 eyes) with primary nasal pterygium presenting to the Regional Institute of Ophthalmology (RIO), Medical College, Kolkata, during the period from January 2018 to June 2019. The study was approved by the Institutional Ethics Committee (IEC No. MC/Kol/IEC/Non-spon/719/12-2017).

The sample size was calculated based on a previous study [6] using the following formula:

$N = \left[ Z_{(1-\alpha/2)} + Z_{(1-\beta)} \right]^2 \times 2\sigma^2 / d^2$

The sample size came to around 37 in each group; after taking 30% errors and rounding off, it came out to around 55 in each group. Therefore, a total of 110 patients were included in our study. Patients aged 18 years or older of either sex, irrespective of race or occupation, with primary progressive pterygium (unilateral or bilateral), and with at least 2 mm of cornea covered by pterygium, were included in the study. We selected the eye with a higher-grade pterygium for patients having bilateral pterygium. Patients with recurrent pterygium, previous intraocular and extraocular surgery, active ocular inflammation, any ocular injury, including physical, chemical, and burn injury, cicatrical ocular surface disease, and corneal scar were excluded from the study.

Grading of the pterygium was done according to the extent of corneal involvement. Grade I, just crossing the limbus; Grade II: midway between limbus and pupil; Grade III: reaching up to the papillary margin; Grade IV: crossing the papillary margin [7].

Informed consent, explaining the purpose and potential risks of the surgical intervention, was obtained from all the patients. All patients were selected after a thorough and relevant ocular and systemic examination preoperatively at the outpatient department (OPD). After full preoperative assessment, 110 patients with primary nasal pterygium were subjected to surgical excision of pterygium. For randomization, the sealed envelope method was used. After randomization, 55 patients were allocated to group A and underwent pterygium excision with the bare sclera technique (BST), and another 55 patients were allocated to group B and underwent pterygium excision with the limbal conjunctival autograft technique (LCAT).

All the surgeries were done under a peribulbar block and after an antiseptic dressing and draping as per norm. After pterygium excision, the bare sclera bed was left behind to re-epithelialize in group A, and after excision, a conjunctival autograft was used over the bare sclera in the correct anatomical position and fixed with 8-0 Vicryl suture with a spatulated needle in group B. To reduce interobserver bias, a single ophthalmologist performed all preoperative assessments and postoperative evaluations, and a single surgeon performed all surgical procedures.

All the patients were assessed at the first OPD visit as preoperative and after two weeks, four weeks, and 12 weeks of surgery as postoperative follow-up visits. All study patients were assessed for visual acuity, with best corrected visual acuity (BCVA) recorded on the LogMAR (Logarithm of the Minimum Angle of Resolution) scale in LogMAR units. Refraction was measured first by autorefractometry and then by subjective refraction in diopters (D). Keratometry was performed using a Bausch and Lomb type keratometer. The findings were compared within and between the groups.

The collected data were entered in Microsoft Excel (Microsoft Corp., Redmond, WA) and analysed using SPSS version 21 (IBM Corp., Armonk, NY). Quantitative data were expressed as means and standard deviations (SD), while qualitative data were expressed in frequencies and percentages. To compare the variables, paired and unpaired t-tests were used. A p< 0.05 was considered statistically significant.

## Results

A total of 110 eyes of 110 patients with primary nasal pterygium were randomly included and divided into two groups, with 55 patients in each group. Out of 110 patients studied, 44 were males (40%) and 66 were females (60%), indicating that pterygium is more common in the female population. Additionally, 77 (70%) of patients fell within the 26- to 50-year age group. The mean age of patients was 41.6 years. Of the patients, 78 (70.9%) had a grade II pterygium, and 32 (29.1%) had a grade III pterygium.

In group A (BST), the preoperative mean BCVA LogMAR score of 0.37±0.15 was significantly changed to LogMAR score 0.24±0.11 (p <0.05), LogMAR score 0.19±0.09 (p <0.001), and LogMAR score 0.15±0.10 (p <0.001) after two weeks, four weeks, and 12 weeks, respectively. Similarly, in group B (LCAT), the preoperative mean BCVA LogMAR score of 0.37±0.17 was significantly changed to LogMAR score 0.19±0.13 (p <0.001), LogMAR score 0.12±0.08 (p <0.001), and LogMAR score 0.08±0.09 (p <0.001) postoperatively after two weeks, four weeks, and 12 weeks, respectively. In group A, the preoperative mean astigmatism of 2.10±0.84 D was significantly reduced to 1.60±0.79 D (p <0.01), 1.47±0.71 D (p <0.05), and 1.30±0.56 D (p <0.001) postoperatively after two weeks, four weeks, and 12 weeks, respectively. Similarly, in group B, the preoperative mean astigmatism of 2.08±0.96 D was significantly reduced to 1.20±0.71 D (p <0.001), 0.89±0.51 D (p <0.05), and 0.70±0.40 D (p <0.001) after two weeks, four weeks, and 12 weeks of surgery, respectively (Table 1).

**Table 1:** Comparison of Pre- and Postoperative Visual Acuity (in LogMAR Score), and Astigmatism (in Diopters). In Mean ± SD *Paired t-test; SD, standard deviation

Variable	Group	Pre-operative	2 weeks	p-value^*^	4 weeks	p-value^*^	12 weeks	p-value^*^
Visual acuity	Group A	0.37±0.15	0.24±0.11	<0.05	0.19±0.09	<0.001	0.15±0.10	<0.001
	Group B	0.37±0.17	0.19±0.13	<0.001	0.12±0.08	<0.001	0.08±0.09	<0.001
Astigmatism	Group A	2.10±0.84	1.60±0.79	<0.01	1.47±0.71	<0.05	1.30±0.56	<0.001
	Group B	2.08±0.96	1.20±0.71	<0.001	0.89±0.51	<0.05	0.70±0.40	<0.001

A significant difference in keratometry findings was observed before and after pterygium excision. In group A, the mean pre-operative dioptric power of the cornea at the horizontal axis (K1) changed from 42.50±0.81 D to 41.40±0.46 D (p<0.001) and at the vertical axis (K2) changed from 41.40±0.83 D to 40.60±0.30 D (p<0.001) after 12 weeks of surgery. Similarly, in group B, the mean preoperative dioptric power of the cornea at the horizontal axis (K1) changed from 42.70±0.55 D to 41.20±0.20 D (p<0.001) and at the vertical axis (K2) changed from 41.80±0.40 D to 40.50±0.20 D (p<0.001) after 12 weeks of surgery (Table 2).

**Table 2:** Comparison of Pre-operative and Post-operative Keratometry Findings (Mean ± SD in Diopters) *Paired t-test; SD, standard deviation

Group	Variable	Pre-operative	Post-operative			Difference at 12 Weeks After Surgery	p-value*
			2 Weeks	4 Weeks	12 Weeks		
Group A	Horizontal (K1)	42.50±0.81	41.80±0.69	41.60±0.56	41.40±0.46	1.10±0.35	<0.001
	Vertical (K2)	41.40±0.83	40.90±0.50	40.70±0.37	40.60±0.30	0.80±0.53	<0.001
Group B	Horizontal (K1)	42.70±0.55	41.80±0.40	41.30±0.30	41.20±0.20	1.50±0.35	<0.001
	Vertical (K2)	41.80±0.40	41.00±0.30	40.60±0.20	40.50±0.20	1.30±0.20	<0.001

The mean visual acuity was better in all postoperative follow-ups in group B than in group A, and the difference between the groups was statistically significant after two weeks (p < 0.05), four weeks (p < 0.001), and 12 weeks (p < 0.001). Post-operative mean astigmatism was lower in group B than in group A in all postoperative follow-ups. The difference in mean astigmatism between the groups was statistically significant after two weeks (p < 0.05), four weeks (p <0.001), and 12 weeks (p < 0.001) (Table 3).

**Table 3:** Comparison Between the Bare Sclera and Conjunctival Autograft Group in Terms of Visual Acuity (in LogMAR Score) and Astigmatism (in Diopters). In Mean ± SD # Unpaired t-test; SD, standard deviation

Visual Acuity (LogMAR)				
Group	Pre-operative	2 Weeks	4 Weeks	12 Weeks
Group A	0.37±0.15	0.24±0.11	0.19±0.09	0.15±0.10
Group B	0.37±0.17	0.19±0.13	0.12±0.08	0.08±0.09
‘p’ value^#^	>0.05	<0.05	<0.001	<0.001
Astigmatism				
Group A	2.10±0.84	1.60±0.79	1.47±0.71	1.30±0.56
Group B	2.08±0.96	1.20±0.71	0.89±0.51	0.70±0.40
‘p’ value^#^	>0.05	<0.05	<0.001	<0.001

In both group A and group B, there was a decrease in with-the-rule astigmatism after each follow-up. The mean diopteric power of the cornea in the horizontal axis (K1) changed from 42.50±0.81 D to 41.40±0.46 D after 12 weeks postoperatively in the bare sclera technique and from 42.70±0.55 D to 41.20±0.20 D after 12 weeks in the conjunctival autograft technique, which was better than in the bare sclera technique. The mean diopteric power of the cornea in the vertical axis (K2) changed from 41.40±0.83 D to 40.60±0.30 D after 12 weeks postoperatively in the bare sclera technique and from 41.80±0.40 D to 40.50±0.20 D after 12 weeks postoperatively in the conjunctival autograft technique, which was better than in the bare sclera technique. The difference in keratometry values after 12 weeks of surgery between the two groups was statistically significant (p<0.05) (Table 4).

**Table 4:** Comparison Between the Bare Sclera and Conjunctival Autograft Group in Terms of Keratometry Findings (Mean ± SD in Diopters). # Unpaired t-test; SD, standard deviation; K1, horizontal axis; K2, vertical axis

Group	Keratometry Findings							
	Pre Operative		2 Weeks		4 Weeks		12 Weeks	
	K1	K2	K1	K2	K1	K2	K1	K2
Group A	42.50±0.81	41.40±0.83	41.80±0.69	40.90±0.50	41.60±0.56	40.70±0.37	41.40±0.46	40.60±0.30
Group B	42.70±0.55	41.80±0.40	41.80±0.40	41.00±0.30	41.30±0.30	40.60±0.20	41.20±0.20	40.50±0.20
‘p’ value^# ^	>0.5	<0.05	>0.05	>0.05	<0.001	>0.05	<0.05	<0.05

## Discussion

There have been previous studies that have reported varying degrees of correction of refractive error with different techniques of pterygium excision. Ours was a prospective comparative study on a total of 110 patients (110 eyes) with primary nasal pterygium. The study patients were randomly distributed into two groups of 55 cases each. Group A was treated by the BST, while group B was treated by the LCAT.

Our study observed a significant reduction in astigmatism and an improvement in visual acuity following pterygium excision using both the BST and LCAT techniques. Similar observations were made by Garg et al. [6], where the mean astigmatism was significantly reduced after three months postoperatively in both techniques. Gahlot et al. [8] verified that pterygium excision with conjunctival autografting could result in a significant decrease in corneal astigmatism and thus improve the visual acuity of patients postoperatively. A similar trend in visual acuity (LogMAR units) and astigmatism was observed in Gangadhar et al. [9]. A similar decrease in astigmatism was also seen in an article by Mohite et al. [10], where pre-operative astigmatism of 3.12 ±1.236 D decreased to 1.536±0.642 D (p<0.001) post-operatively. In our study, a significant difference in keratometry findings was observed before and after pterygium excision in both BST and LCAT groups. A similar paper observed significant improvement in the mean BCVA from 6/7.5 preoperatively to 6/6 at 1 month (p value 0.001) after pterygium surgery [11].

In our study, the mean visual acuity was better in all post-operative follow-ups in group B than in group A, and the difference between the groups was statistically significant after 12 weeks of surgery (p <0.001). In our study, post-operative mean astigmatism was lower in group B than in group A in all postoperative follow-ups. We observed that the difference in mean astigmatism between the groups was statistically significant after 12 weeks (p<0.001) of surgery. A similar observation was reported in Garg et al. [6], in which the three-month postoperative changes in astigmatism in the bare sclera and conjunctival autografting groups were from 3.43±1.73 D to 1.58±0.95 D and from 3.50±1.77 D to 0.95±0.60 D, respectively, and both were statistically significant. Their study also showed a significant difference in the astigmatic changes between the bare sclera and the conjunctival autograft technique (p-value 0.035). Another study [12] also found that the successful pterygium excision and limbo-conjunctival autograft surgery significantly reduces topographic astigmatism, which corroborates our research findings. A similar study [13] compared the astigmatism changes by using the bare sclera technique and the conjunctival autograft technique. They found that the decrease in astigmatism after pterygium surgery was greater in the conjunctival autograft technique (6.21 D to 1.0 D) as compared to the bare sclera technique (6.17 D to 2.85 D) after six weeks post-surgery, and concluded that the type of pterygium surgery also plays a role in modifying the pterygium-induced astigmatism.

In our study, we found an improvement in the rule astigmatism in both groups, and the improvement was better in group B than in group A, and the difference between groups was statistically significant (p<0.05) after 12 weeks of surgery. In contrast to our study findings, Sen et al. [14] observed that the astigmatic change was insignificant in the conjunctival autograft group using suture after three months postoperatively, and an insignificant difference was found in horizontal keratometric value (p-value 0.6289) and vertical keratometric value (p-value 0.9461) at the end of three months. Altan-Yaycioglu et al. [15] also concluded that the type of grafting or the use of suture to fixate the graft does not have a significant effect on astigmatism.

Our cohort was assessed for a short-term follow-up period of 12 weeks after surgery. Therefore, our research was limited in terms of evaluating long-term recurrence, as it is an important parameter in choosing one surgical technique over another. So, we recommend that further studies with a longer follow-up period be conducted.

## Conclusions

Pterygium excision results in a significant improvement in visual acuity, astigmatism, and keratometry values in both techniques progressively in all postoperative follow-ups. There was a significant difference in visual acuity, astigmatism, and keratometry values between the bare sclera technique (group A) and the limbal conjunctival autograft technique (group B) after all postoperative follow-up visits, and the conjunctival autograft was found to be better than the bare sclera. Therefore, our findings indicate that the conjunctival autograft technique is preferable to the bare sclera technique in improving visual acuity and reducing astigmatism.

## References

[1] Zloto O, Barequet I, Ezra Nimni O, Berger Y, Gildener-Leapman J, Antman G, Avni-Zauberman N (2023). The change in corneal and conjunctival sensation following pterygium surgery. Isr Med Assoc J.

[2] Clearfield E, Muthappan V, Wang X, Kuo IC (2016). Conjunctival autograft for pterygium. Cochrane Database Syst Rev.

[3] Sharma B, Bajoria SK, Mishra M, Iqubal N (2021). Refractive outcomes of simultaneous pterygium and cataract surgery with fibrin glue. Cureus.

[4] Singh SK (2017). Pterygium: epidemiology prevention and treatment. Community Eye Health.

[5] Oh JY, Wee WR (2010). The effect of pterygium surgery on contrast sensitivity and corneal topographic changes. Clin Ophthalmol.

[6] Garg P, Sahai A, Shamshad MA, Tyagi L, Singhal Y, Gupta S (2019). A comparative study of preoperative and postoperative changes in corneal astigmatism after pterygium excision by different techniques. Indian J Ophthalmol.

[7] Maheshwari S (2003). Effect of pterygium excision on pterygium induced astigmatism. Indian J Ophthalmol.

[8] Gahlot A, Maheshgauri RD, Kumari P, Datta D (2015). Comparison of pre and post operative corneal astigmatism following pterygium excision and conjunctival autograft. J Med Sci Clin Res.

[9] Gangadhar DP, Shankar KH, Choudhary KG (2014). Effect of pterygium surgery by using conjunctival autograft and amniotic membrane graft on astigmatism and visual acuity. MedPulse - International Medical Journal.

[10] Mohite US, Dole NB, Jadhav SS (2017). Effectiveness of pterygium surgery on corneal astigmatism. Med Pulse Int J Ophthalmol.

[11] Misra S, Craig JP, McGhee CN, Patel DV (2014). A prospective study of pterygium excision and conjunctival autograft with human fibrin tissue adhesive: effects on vision, refraction, and corneal topography. Asia Pac J Ophthalmol (Phila).

[12] Errais K, Bouden J, Mili-Boussen I, Anane R, Beltaif O, Meddeb Ouertani A (2008). Effect of pterygium surgery on corneal topography. Eur J Ophthalmol.

[13] Popat KB, Sheth HK, Vyas VJ, Rangoonwala MM, Sheth RK, Shah JC (2014). A study on changes in keratometry readings and astigmatism induced by pterygium before and after pterygium excision surgery. J Res Med Den Sci.

[14] Sen S, Jain A, Upadhyay P, Imchen MT, Nath T, Kakkar A (2023). Comparison of keratometric change following various conjunctival autografting techniques in pterygium surgery. Cureus.

[15] Altan-Yaycioglu R, Kucukerdonmez C, Karalezli A, Corak F, Akova YA (2013). Astigmatic changes following pterygium removal: comparison of 5 different methods. Indian J Ophthalmol.

